# How to Preserve Vaccine Matter

**Published:** 1865-04

**Authors:** D. Prince

**Affiliations:** Jacksonville, Ills.


### HOW TO PRESERVE VACCINE MATTER.

By Dr. D. PRINCE, of Jacksonville, Ills.

Take a portion of common alum (Sulph. Alumina and Potassa) and boil it upon a hot iron—a red-hot shovel is generally employed—until the water of crystalization is driven out. From this exsiccated alum, fill half-full a bottle of an ounce or two capacity; such a bottle as is usually employed for Sulphate of Morphia is convenient for the purpose. Envelope the vaccine scab in a convenient fold of paper and place in the vial over the alum. Adapt a well fitting cork, cut off even with the top of the bottle; seal air tight with good sealing wax, and set the bottle away upon a shelf.

The one indispensible requisite to the preservation of Vaccine matter is to dry it completely and keep it dry. There is probably hardly any limit to the time it may be kept perfectly fresh and susceptible, if this condition is complied with. Any other substance having a strong affinity for water like fresh-burned lime would do well, but alum, on the whole, most perfectly combines cheapness and convenience. The writer has kept Vaccine matter through two hot seasons by this method, and has acquired entire confidence in the method.

The same material may be used over from year to year by re-heating, to be sure that the vapor absorbed is thoroughly expelled. Upon this condition depends the success of the method.

For perfect safety in transmission, the bottle thus prepared should be enveloped in a tin case, which should be soldered air tight. Thus prepared, the package might be sent round the world with perfect confidence in the preservation of the virtues of the matter.

The method of enveloping the *undried* scab, or the lymph preserved upon quill points, in wax or tin foil, or sealing them in glass tubes, without drying material to absord the moisture both of the matter and of the air, is worthy of no confidence, and indeed is utterly useless.

Jacksonville, March 15, 1865.

